# Log::ProgramInfo: A Perl module to collect and log data for bioinformatics pipelines

**DOI:** 10.1186/s13029-016-0055-9

**Published:** 2016-06-24

**Authors:** John M. Macdonald, Paul C. Boutros

**Affiliations:** Informatics and Biocomputing Program, Ontario Institute for Cancer Research, Suite 510, MaRS Centre, 661 University Ave, Toronto, Ontario Canada; Departments of Medical Biophysics and Pharmacology & Toxicology, University of Toronto, Toronto, Ontario Canada

**Keywords:** Reproducibility, Log, Environment

## Abstract

**Background:**

To reproduce and report a bioinformatics analysis, it is important to be able to determine the environment in which a program was run. It can also be valuable when trying to debug why different executions are giving unexpectedly different results.

**Results:**

Log::ProgramInfo is a Perl module that writes a log file at the termination of execution of the enclosing program, to document useful execution characteristics. This log file can be used to re-create the environment in order to reproduce an earlier execution. It can also be used to compare the environments of two executions to determine whether there were any differences that might affect (or explain) their operation.

**Availability:**

The source is available on CPAN (Macdonald and Boutros, Log-ProgramInfo. http://search.cpan.org/~boutroslb/Log-ProgramInfo/).

**Conclusion:**

Using Log::ProgramInfo in programs creating result data for publishable research, and including the Log::ProgramInfo output log as part of the publication of that research is a valuable method to assist others to duplicate the programming environment as a precursor to validating and/or extending that research.

## Background

Reproducibility is a major concern in science as a whole, and computational biology in particular. For reproducibility, it is not sufficient to provide access to the raw data—it is ever more critical to also provide access to the program code used to analyse those data [2]. But *the program code* is a dynamic mixture of program text, command line arguments, libraries, and various other environmental aspects—all of which may need to be exactly reproduced to achieve the same results. So, simply providing access to the code used is not a complete solution. It is necessary, but not sufficient.

The need for reproducibility is growing because our pipelines are getting increasingly complex: a typical sequencing pipeline might involve a chain of a dozen unique tools [3]. But reproducing these pipelines is fundamentally very difficult, in part because it requires duplicating the versions of all dependent tools and libraries used in an analysis. Given the rapid rate of release of updates to common tools (e.g. BWA had 7 updates during the course of 2014 [4], this can be a significant challenge.

Among the best practices for scientific computing (e.g. [5]) is listed the need to collect and publish:

- Unique identifiers and version numbers for programs and libraries;
- The values of parameters used to generate any given output; and
- The names and version numbers of programs (however small) used to generate those outputs.

A large fraction of pipelines for bioinformatics are written in the Perl programming language (e.g. BioPerl [6]). However, for logging the precise state of a program at run-time, and capturing all the dependency versions and other key information, there are no automated choices available.

To resolve this issue, we introduce here the module Log::ProgramInfo to facilitate run-time logging of Perl-based pipelines, thereby directly improving the reproducibility of modern bioinformatic analyses.

A further advantage to such tracking information is the ability to test an analsis using later versions of the component tools to determine whether they provide different results (possibly more accurate if the later releases provide better resolution; possibly identifying erroneous results in the original analysis if the tools have been updated with critical fixes to their operation).

## Related work

A search found some programs for related processes but nothing that served the same purposes.

There are some programs available to collect and document the computing process - by recording the steps invoved, including command lines and arguments during the actual data processing. Such a program could work well together with the described module but addresses a different aspect of the reproducibility issue. In our lab, when the workflow of the data analysis was sufficiently complex to require such a description, we instead write a program to encapsulate that process, so there is no long list of manual processing steps to document.

In particular, the program (ReproZip) [7] was capable of discovering and bundling together all of the programs used during the execution of a process. That seems to have different trade-offs. Such a bundle is only useful on similar hardware and it provides no possibility for assisting with script library version info, or in allowing a later run to use selected variations on the programming environment (such as allowing updated versions of programs that still have the same function but have had security problems fixed).

## Implementation

The Log::ProgramInfo module Macdonald and Boutros, Log-ProgramInfo. http://search.cpan.org/~boutroslb/Log-ProgramInfo/ is available as open source, and has been distributed on CPAN (the Comprehansive Perl Archive Network - used as the standard distribution mechanism for the vast majority of open source Perl modules, and described in the Perl documentation with the command “perldoc perlmodinstall”).

Log::ProgramInfo is enabled simply by being included with a Perl **use** statement. Since its effect is global to the program, it should be enabled directly from the main program, or from a utility module that contains global configuration settings for a suite of programs.

Any desired setting of non-default values for the options can be provided either through environment variables, or as “import” list options.

When the module is used for the first time, the loading process carries out a number of actions for its operation:

- An END block is created. It will be executed when the program terminates, to write out the log information.
- Signal handlers are installed for catcheable signals - if one of them occurs, the log information will be printed out before the program terminates.
- options are set to their default values
- any env variables to control options are saved
- a copy is made of the original command line arguments for eventual logging
- the start time is recorded for eventual logging
-... (numerous other system attributes are saved for eventual logging)

Every time the Log::ProgramInfo module is used, the import list is processed and any values in it are used to update the option values. (The first time it is used, this processing happens **after** the initialization steps described above.)

That permits a common group of option settings be processed first, and then specific exceptions to that list over-ridden.

Any option settings provided in environent variables will over-ride the corresponding setting (whether a default or specified by the program import lists). This allows changing the option settings for individual runs so that the log can be suppressed, enabled, or redirected for a single run of the program.

The code that prints the log information ensures that it only executes once (in case multiple signals, or a signal during program termination, would cause it to be called additional times).

If the main body of the program changes a signal handler after Log::ProgramInfo has set it up, that will usually not interfere with Log::ProgramInfo. Usually, the program will catch signals and handle them in a way that allows it continue to operate, or to terminate with an exception. It is only if the program resets a signal handler to its default (abort without normal termination processing) that Log::ProgramInfo’s log will not be written. That is not a problem for publication - if the program is being killed by some signal then it is not yet running successfully, and thus not yet ready for publication. However, it does mean that the log might not be available as a diagnostic aid in such situations.

For most cases, that is the only interaction between the program and Log::ProgramInfo.

The one additional interaction that might occur is if there is information unique to the program that is desired to be logged. The function

**Log::ProgramInfo::add_extra_logger** can be called by the program to specify a callable function that will write additional information to the log. (See the program documentation for precise details.)

## Results and discussion

Parameters are available to control the logging process: whether (and if so, where) a log is to be written. Choosing the location where the log is written allows collecting and managing this important information in a way that co-ordinates with the entire set of computational activity carried out for a research project (or an entire organisation’s collection of research projects). The default name used for the log file includes the name of the program that is being reported upon as well as a time-stamp to distinguish separate runs—you might choose to override the name or directory path to provide more complete organisation of logged results. Suppressing log output can be useful for runs that are not intended to generate reproducible results, such as while the software is being developed. However, even in such cases, it might turn out to be useful to have this log output to assist diagnosing problems with system configuration changes—to confirm that the environment being used is the one that was intended and that updates have actually occurred, etc.

There is an additional parameter that permits the logged information to be sent to a separate logging mechanism, such as a Log4Perl log. This would allow the information to be collected with the other logged information from the program. The output to such logs is mixed with the other logged output from the program, and is also usually reformatted to some extent. Such logs cannot be processed by the Log::ProgramInfo parser provided with the package; hence the normal action for Log::ProgramInfo is to still write its own log file as well.

## Log output

The output created by Log::ProgramInfo contains the following information:

- **MODULE** – Name, version, file location, and checksum for each perl library module used by the program.
- **INC** – The search path used to find modules.
- **UNAME** – Operating system information.
- **PROCn** – Specific information for each processor (memory, cores, etc.)
- **PERL** – The perl interpretor pathname.
- **PERLVer** – The perl interpretor version.
- **PERLSum** – Checksum of the perl interpretor binary.
- **libc** – The version of libc used by the perl interpretor.
- **libcSUM** – Checksum of the libc library used by the perl interpretor.
- **User** – The user ID (and real user ID, if different) running the program.
- **Group** – The group IDs (and real group IDs, if different) running the program.
- **ProgDir** – The directory containing the program.
- **Program** – The program name.
- **Version** – The program’s version.
- **ProgSUM** – Checksum of the program file.
- **Args** – The number and values of the command line arguments provided to the program.
- **Start** – The time the program started running.
- **End** – The time the program stopped running.
- **Elapsed** – The elapsed time while the program was running.
- **EndStat** – The program’s exit status.
- program-specified – Any additional info provided by program-specified callback functions.

The format of the log file is designed to be easily parsed. A parsing subroutine is provided in the package. You could call that subroutine from a program that analyses logs according to your needs. See the program documentation for details. If you have written the log info using a logging module such as Log4Perl, you will have to separately extract the bare ProgramInfo log information out of that log, separating it from any other logging by the program, and removing any line decorations added by the log module.

## Example

Here is an example of using Log::ProgramInfo. Assume a simple program, called simple.pl.



When you run it, you get two lines of output.



The first line is the expected output from the program, the second line comes from Log::ProgramInfo to tell you that a log file was created, and where.

Now, take a look at the log file:

- lines beginning with a plus sign are wrapped to fit the page width
- lines wrapped in angle brackets describe text that has been omitted for brevity

Now that you have a log file, you still have to make use of it. Typically, you would treat this log file as one of the output files of your processing activities. So, if you normally discard the output files (e.g. for a test run while developing the pipeline), you will likely also discard the log. On the other hand, for significant runs, you would collect the log file along with the other output files, labelling and storing them as appropriate for reference. The log file would be available as a synopsis of how the output data was created, ready to be used for publication, or reproducing the process (either to validate the results, or to apply the same process to additional data for subsequent research).

## Limitations

The C environment is not well built for program introspection activities such as determining which static and/or dynamic libraries have been linked into the program’s executable image. This module lists the version of libc that was build into the perl binary - but that information can be out of date. A future release may try to get info about other libraries beyond libc.

Another major problem is that even if a perl module is downloaded from CPAN (which would be one way of ensuring that other people could get the same version), the install process that puts it into the library path for perl programs can be done in may ways, and often is not even done on the same computer as the one that is running the perl program. So, it is not easy to do any sort of detailed validation - the downloaded package bundle is not accessible in any determinable way (and possibly not at all) to the program itself (and thus to Log::ProgramInfo). While it would be possible to compute checksums for every library module that has been loaded, that would take a significant amount of time and is not currently being done. It may be added as an option that could request it explicitly.

## Conclusion

Module Log::ProgramInfo provides a convenient way of logging information about the way a program is run. Adding it to existing programs is as easy as adding one line to the program or any module the program already includes.

Log::ProgramInfo’s output file can be easily included in the published results along with the actual source code (or references to where it can be found). With this log output, other researchers have information necessary to any meaningful attempt to reproduce the original research, either in the process of validating or extending that research.

Log::ProgramInfo is a good candidate for inclusion in modules intended to mandate standards, and may find use well beyond the field of bioinformatics.

## Availability and requirements

- **Project name:** LogProgramInfo
- **Project Home Page:**http://search.cpan.org/search?query=Log%3A%3AProgramInfo&mode=all
- **Operating System(s):** Linux, Unix, Mac OS X (untested), Windows (untested)
- **Programming Language:** Perl 5
- **Other Requirements:** none
- **License:** Perl 5 License (Artistic 1 & GPL 1)
